# Distinct Genetic Alterations in Colorectal Cancer

**DOI:** 10.1371/journal.pone.0008879

**Published:** 2010-01-26

**Authors:** Hassan Ashktorab, Alejandro A. Schäffer, Mohammad Daremipouran, Duane T. Smoot, Edward Lee, Hassan Brim

**Affiliations:** 1 Department of Medicine and Cancer Center, Howard University, College of Medicine, Washington, DC, United States of America; 2 National Center for Biotechnology Information, National Institutes of Health (NIH), Department of Health and Human Services (DHHS), Bethesda, Maryland, United States of America; 3 Department of Pathology, Howard University, College of Medicine, Washington, DC, United States of America; Dr. Margarete Fischer-Bosch Institute of Clinical Pharmacology, Germany

## Abstract

**Background:**

Colon cancer (CRC) development often includes chromosomal instability (CIN) leading to amplifications and deletions of large DNA segments. Epidemiological, clinical, and cytogenetic studies showed that there are considerable differences between CRC tumors from African Americans (AAs) and Caucasian patients. In this study, we determined genomic copy number aberrations in sporadic CRC tumors from AAs, in order to investigate possible explanations for the observed disparities.

**Methodology/Principal Findings:**

We applied genome-wide array comparative genome hybridization (aCGH) using a 105k chip to identify copy number aberrations in samples from 15 AAs. In addition, we did a population comparative analysis with aCGH data in Caucasians as well as with a widely publicized list of colon cancer genes (CAN genes). There was an average of 20 aberrations per patient with more amplifications than deletions. Analysis of DNA copy number of frequently altered chromosomes revealed that deletions occurred primarily in chromosomes 4, 8 and 18. Chromosomal duplications occurred in more than 50% of cases on chromosomes 7, 8, 13, 20 and X. The CIN profile showed some differences when compared to Caucasian alterations.

**Conclusions/Significance:**

Chromosome X amplification in male patients and chromosomes 4, 8 and 18 deletions were prominent aberrations in AAs. Some CAN genes were altered at high frequencies in AAs with *EXOC4, EPHB6, GNAS, MLL3* and *TBX22* as the most frequently deleted genes and *HAPLN1, ADAM29, SMAD2* and *SMAD4* as the most frequently amplified genes. The observed CIN may play a distinctive role in CRC in AAs.

## Introduction

Colorectal cancer is the third most common cancer in the United States [1]. It has a higher incidence and causes more deaths in African Americans than in other racial groups. Most colorectal cancers arise from adenomas, in a process described as the adenoma-carcinoma sequence [2]. Like other cancers, initiation and progression of CRC are associated with an accumulation of alterations in the function of key regulatory genes and genetic instability.

Three major forms of genetic instability in CRC have been described [2], [3], [4]. In about 13% of CRC cases, mismatch repair deficiency leads to microsatellite instability (MIN)[5]. Approximately 40% of CRC tumors are characterized by epigenetic changes especially DNA methylation, a phenomenon termed CpG Islands Methylator Phenotype (CIMP) [6], [7]. In the remaining 47% of CRCs, chromosomal instability leads to gains and losses of large segments of chromosomes [8].

The CIN category includes cancers with aneuploid or polypoid karyotypes, and cancers that have multiple gains or deletions of chromosomal arms, or multiple translocations. CIN results from specific mutations and/or genes being silenced and could result from structural defects involving centromeres or centrosome, microtubule dysfunction, telomeres erosion, chromosome breakage and failure of cell cycle checkpoints [9]. The acquisition of recurrent chromosomal gains and losses during the progression from high-grade adenomas to invasive carcinomas is found in CRC tumors [10]. One of the earliest acquired genetic abnormalities during CRC progression involves chromosome 7 amplification, which is also observed in some colon adenomas [11]. At later stages, other specific chromosomal aberrations become common, such as gains on 8q, 20q [12], 7, 13 [13], [14] and deletions on 8p, 17p, 18q [13], [15] 15q and 20q [16].

CIN and MIN phenotypes were initially considered mutually exclusive since MIN tumors generally have stable and diploid karyotypes [17], [18]. However, recent studies have found that MIN and CIN can occur in the same tumor [19], [20]. Trautmann et al. found that at least 50% of MSI-H tumors have some degree of chromosomal alterations [21]. Although evidence for some degree of CIN could be found in the majority of MSI-H tumors, the specific alterations identified differed between MSI-H and MSS tumors. MSI-H tumors harbor gains of chromosomes 8, 12, 13 and losses of 15q and 18q while MSS tumors have a high degree and variable chromosomal range of aberration [16], [21].

Lassmann et al. studied 287 target sequences in 22 Caucasian colorectal tumors and found frequent aberrations in specific regions of chromosomes 7, 8, 13, 17, 20 and suggested some candidate genes with frequent deletion or amplification in these regions [22]. Studies that identify genes with altered copy number associated with tumorigenesis may lead to the detection of specific targets for cancer therapy and increase our understanding of tumorigenesis. We hypothesize that identification of chromosomal aberrations in CRCs from AA patients may help explain aspects of colon cancer pathogenesis specific to this population. Therefore, we investigated the CIN in AA CRC patients by applying aCGH to tumor samples. We compared our results with the recently published findings in Caucasians [22] and with a list of colon cancer genes proposed by Sjöblom et al [23] after their thorough genetic analysis of 11 colon tumors.

## Materials and Methods

### Ethics Statement

This study was approved by Howard University Institutional Review Board, and written informed consent was obtained.

### Patients

Fresh frozen colonic biopsies (n = 15) were obtained from African-American patients undergoing colonoscopy at Howard University Hospital. This study was approved by the Howard University Institutional Review Board. The purpose of this study was explained to the patients before colonoscopy and the participating patients gave informed consent. Clinical data collected on each patient included race, gender, associated past medical history, medication use, and family history of colonic cancer. Patients were deemed eligible if colonoscopy resulted in a diagnosis of adenocarcinoma, confirmed by histopathology. From the review of medical records, clinical information was collected and recorded based on the American Joint Committee on Cancer staging system. Patients in this study self-identified as AAs.

### Samples Selection and DNA Extraction for aCGH Analysis

Fresh tumor blocks were cut into 5 µm sections on Superfrost slides (Fisher Scientific, Pittsburgh, PA). The tumor and normal areas were distinguished by a pathologist (E.L) using the H&E matched slide and microdissected to pinpoint the tumor as well as normal areas. Tumor and normal corresponding areas from fresh frozen samples were used for DNA extraction using Puregene kit (San Francisco, CA) according to the manufacturer's instructions. The goal of the microdissection was to avoid the cross contamination of normal and tumor tissues which would impact the outcome of the aCGH experiment.

### aCGH Experiments and Statistical Data Analysis

In this experiment, we studied the profile of chromosome aberrations in 15 colon adenocarcinomas. Our reference controls were either matched normal or sex-matched normal DNA with no history of any disease to determine the impact of chromosomal aberrations by aCGH in AA colon adenocarcinomas. The colon tissues were evaluated by a GI pathologist for proper histological features that were used for this study including the size, type, location and pathological criteria of the carcinomas. An oligo microarray-based CGH using a chip containing 105,000 human probes (Agilent, Santa Clara, CA) was used. The carcinomas were defined histologically and then after confirmation by the pathologist, we used the corresponding fresh–frozen tissue.

For each aCGH experiment, 1.5 µg of reference DNA and 1.5 µg of one tumor DNA were used. Briefly, the test and reference DNAs were digested with *Alu* I and *Rsa* I (Promega, Madison, WI), and purified with the QIAprep Spin Miniprep kit (QIAGEN, Germantown, MD). Test DNA (1.5 µg) and reference DNA (1.5 µg; Promega) were labeled by random priming with Cy5-dUTP and Cy3-dUTP, respectively, using the Agilent Genomic DNA Labeling Kit Plus. Following the labeling reaction, the individually labeled test and reference samples were concentrated using Microcon YM-30 filters (Millipore, Billerica, MA) and then combined. Following probe denaturation and pre-annealing with Cot-1 DNA, hybridization was performed at 65°C with rotation for 40 hr at 20 rpm. Four steps were done with Agilent Oligo CGH washing solutions: wash buffer 1 at room temperature for 5 min, wash buffer 2 at 37°C for 1 min, an acetonitrile rinse at room temperature for 1 min and a 30 sec wash at room temperature in Agilent's Stabilization and Drying Solution. Copy number variations (CNVs) were identified by Agilent Feature Extraction software 9 and analyzed with Agilent CGH analytics 3.4 software, using the statistical algorithms z score and ADM-2 using sensitivity thresholds of 2.5 and 9, respectively and a moving average window of 0.2 Mb. Locations of CNVs were reported with respect to the human genome sequence assembly Build 35, Hg17 (www.ncbi.nlm.nih.gov).

### Analysis of Gene Content of CNVs

Names of genes suggested in [22], [23] were standardized using the HUGO Gene Nomenclature Committee web site (www.genename.org). Positions of these genes were determined using the data files underlying NCBI's MapViewer browser (www.ncbi.nlm.nih.gov/mapview). Using software newly developed for this study, we identified each overlap between candidate genes and CNVs such that the gain or loss ratio was more extreme than a user-specified threshold for the ratio. For the results shown below, we used the thresholds of ≥1.2 and ≤0.8 for gains and losses, respectively. The software takes as inputs:

1. A file of genes with the chromosome, start position, and end position of each gene;
2. A list of CNVs specifying the start, end, and ratio of each gene;
3. Thresholds for gains and losses.

The software reports every gene such that there is an intersecting gain/loss whose ratio is above/below the user-specified ratios. For example the gene *APC* is located on human chromosome 5 in the interval [112101484, 112209836]. Two patients have losses with ratios <0.8 intersecting the interval [112101484, 112209836] and seven patients have gains with ratios >1.2 intersecting the same interval. The same thresholds were used in the study [22], with which we compared our results. Summary statistics on the amplifications and deletions were tabulated within the new software and using Excel.

## Results

### Characteristics of the Analyzed Samples

We studied 15 colon adenocarcinomas from AA patients. The mean age of this group of patients was 63.5 years with eight females and seven males. The tumors were mainly moderately differentiated (93%), and in stage II or III (87%). Two thirds of the samples were right sided (67%; Table 1). A comparison of our group of patients with those in Lassmann et al [22] has shown no statistically significant differences between the two groups (Table 1).

**Table 1:** Clinical and demographic characteristics of the 15 patients enrolled in this study.

Case number	Age	Sex	Stage	Location	Differentiation
1	53	Female	4	Right Colon	Moderately
2	51	Female	2a	Left Colon	Moderately
3	65	Male	1	Right Colon	Moderately
4	71	Male	2b	Left Colon	Moderately
5	69	Female	2a	Left Colon	Moderately
6	65	Female	2a	Left Colon	Moderately
7	57	Male	3b	Right Colon	Moderately
8	65	Male	3b	Left Colon	Well
9	68	Female	3c	Right Colon	Moderately
10	64	Male	3c	Right Colon	Moderately
11	83	Female	3c	Right Colon	Moderately
12	73	Female	3c	Right Colon	Moderately
13	61	Male	3a	Right Colon	Moderately
14	54	Female	2a	Right Colon	Moderately
15	53	Male	3c	Right Colon	Moderately

### Summary of Genomic Alterations

All 15 cases displayed some chromosomal instability. These chromosomal aberrations were not equally distributed over all the chromosomes. Only chromosome 21 did not show any amplifications and only chromosome 8 did not show any deletions. A total of 182 amplification events and 101 deletion events were found in the 15 samples all together, with mean counts of 12.1 amplifications and 6.7 deletions per patient. average of 20 aberrations were found in each patient on this study. Amplifications were prominent in chromosomes 2 (40%), 6 (47%), 7 (80%), 8 (60%), 12 (40%), 13 (60%), 16 (47%), 20 (67%) and X (60%). Chromosomal deletions were more frequent on chromosomes 2 (47%), 4 (53%), 5 (60%), 7 (40%), 8 (67%), 17 (40%), 18 (60%), 19 (40%), and 22 (40%; Table 2).

**Table 2:** Number and frequency of aberrations per chromosome.

Chromosomes	Amplifications (cases)*	Frequency (%)	Deletions (cases)*	Frequency (%)
1	3 (3)	20	8 (5)	33.3
2	8 (6)	40	8 (7)	46.6
3	5 (4)	26.6	10 (4)	26.6
4	6 (5)	33.3	9 (8)	53.3
5	8 (4)	26.6	12 (9)	60
6	8 (7)	46.6	10 (5)	33.3
7	12 (12)	80	11 (6)	40
8	10 (9)	60	14 (10)	66.6
9	3 (3)	20	0	0
10	6 (5)	33.3	7 (5)	33.3
11	3 (3)	20	5 (5)	33.3
12	11 (6)	40	4 (4)	26.6
13	9 (9)	60	4 (2)	13.3
14	3 (1)	6	3 (3)	20
15	5 (4)	26.6	6 (5)	33.3
16	7 (7)	46.6	2 (2)	13.3
17	6 (6)	26.6	6 (6)	40
18	5 (4)	26.6	12 (9)	60
19	4 (4)	26.6	6 (6)	40
20	10 (10)	66.6	3 (3)	20
21	0	0	5 (5)	33.3
22	1 (1)	6	6 (6)	40
X	10 (9)	60	3 (3)	20
Y	2 (2)	28.6	3 (3)	42.8

*3(3) means 3 amplification in 3 cases; 8 (5) means 8 deletions in 5 cases from total of 15 for the calculated frequency.

The aberrations were unequally distributed among the 15 patients with four patients having less than 10 aberrations (patients 3, 11, 12 and 13). The number of aberrations does not seem to be stage-specific since these four patients are at stages 1, 3c, 3c, and 3a respectively. Other patients at these stages displayed many more aberrations (patients 2, 4, 5, 8, 9, 10, 11 and 13). The number of CNVs does not seem to depend on the age of the patients since patients 1 and 2, the youngest in our group (51 and 53 years old), have 55 and 42 aberrations respectively, while patients 3, 11, 12 and 13 with the fewest number of aberrations are 65, 83, 73 and 61 years old, respectively. Also, there was no correlation between gender and the frequency of CNVs.

### Comparison of the aCGH Data with the CRC CAN Genes

An analysis by Sjöblom et al. [23] of 11 breast and 11 colon tumors led to the establishment of a list of potentially altered genes in these kinds of tumors. More than 90% of the tumors were stage IV in Sjöblom et al compared to 6.6% in this study. We checked our aCGH data for gains/losses of the 68 genes that were found to be generally altered in colon cancer tumors. Most of these genes show a non-zero frequency of alteration in our samples. Among the CRC genes established by Sjöbolm et al [23], the most commonly deleted in our samples were *EPHB6, EXOC4 (SEC8L1), GNAS, MLL3,* and *TBX22*. The most commonly amplified genes were *HAPLN1(CRTL1), ADAM29, SMAD2,* and *SMAD4* (Table 3).

**Table 3:** Status of the 68 genes from Sjöblom et al. list (23) in the subjects of this study.

Gene	Chromosome band	Deleted n (%)	Amplified n (%)
*ABCA1*	9q31.1	4 (26)	0 (0)
*ACSL5*	10q25	1 (6)	3 (20)
*ADAM29*	4q34	0 (0)	8 (53)
*ADAMTS15*	11q25	2 (13)	2 (13)
*ADAMTS18*	16q23	7 (46)	3 (20)
*ADAMTSL3*	15q25.2	2 (13)	3 (20)
*APC*	5q22	2 (13)	7 (46)
*C10orf137*	10q26.1	1 (6)	2 (13)
*C15orf2*	15q11	0 (0)	5 (38)
*CD109*	6q13	5 (38)	3 (20)
*CD248*	11q13	5 (38)	3 (20)
*CD46(MCP)*	1q32	2 (13)	0 (0)
*CHL1*	3p26.1	3 (20)	0 (0)
*CNTN4*	3p26	2 (13)	1 (6)
*CSMD3*	8q23.3	9 (60)	0 (0)
*EPHA3*	3p11.2	2 (13)	2 (13)
*EPHB6*	7q34	12 (80)	0 (0)
*ERCC6*	10q11.2	4 (26)	2 (13)
*ERGIC3(SBDCAG84)*	20q12	10 (66)	1 (6)
*EVL*	14q32.2	1 (6)	3 (20)
*EXOC4(SEC8L1)*	7q31	12 (80)	0 (0)
*EYA4*	6q23	3 (20)	4 (26)
*FBXW7*	4q31.3	0 (0)	5 (38)
*GALNS*	16q24.3	4 (26)	3 (20)
*GNAS*	20q13.3	10 (66)	1 (6)
*GUCY1A2*	11q22	4 (26)	3 (20)
*HAPLN1*	5q14.3	1 (6)	7 (46)
*HIST1H1B*	6p22	8 (53)	1 (6)
*KCNQ5*	6q14	5 (38)	3 (20)
*KIAA1409*	14q32.1	0 (0)	2 (13)
*KRAS*	12p12.1	6 (40)	0 (0)
*KRT73(K6IRS3)*	12q13.3	5 (38)	1 (6)
*LGR6*	1q32.1	2 (13)	1 (6)
*LMO7*	13q22.2	10 (66)	2 (13)
*LRP2*	2q31	4 (26)	1 (6)
*MAP2*	2q34-35	4 (26)	1 (6)
*ACTL9*	19p13.2	4 (26)	6 (40)
*MKRN3*	15q11	0 (0)	4 (26)
*MLL3*	7q36.1	12 (80)	0 (0)
*MMP2*	16q12-13	8 (53)	4 (26)
*NF1*	17q11.2	8 (53)	2 (13)
*OBSCN*	1q42.1	2 (13)	1 (6)
*P2RX7*	12q24	6 (40)	2 (13)
*P2RY14*	3q25	5 (38)	0 (0)
*PHIP*	6q14	5 (38)	3 (20)
*PKHD1*	6p12.2	7 (46)	3 (20)
*PKNOX1*	21q22.3	0 (0)	5 (38)
*PRKD1*	14q11	0 (0)	1 (6)
*PTPRD*	9p23-24	4 (26)	0 (0)
*PTPRU*	1p35	3 (20)	6 (40)
*RET*	10q11.2	3 (20)	1 (6)
*RUNX1T1*	8q22	9 (60)	0 (0)
*SCN3B*	11q23.3	4 (26)	2 (13)
*SFRS6*	20q13.1	10 (66)	1 (6)
*SLC29A1*	6p21	9 (60)	4 (26)
*SLC44A4(C6orf29)*	6p21.3	8 (53)	1 (6)
*SMAD2*	18q21.1	0 (0)	9 (60)
*SMAD3*	15q22.3	2 (13)	3 (20)
*SMAD4*	18q21.1	0 (0)	9 (60)
*SYNE1*	6q25	3 (20)	4 (26)
*TBX22*	Xq21.1	11 (73)	3 (20)
*TCF7L2*	10q25.3	1 (6)	3 (20)
*TGFBR2*	3p22	2 (13)	0 (0)
*TP53*	17p13.1	4 (26)	7 (46)
*TTLL3*	3p25.3	2 (13)	0 (0)
*UHRF2*	9p24.1	4 (26)	0 (0)
*UQCRC2*	16p12	8 (53)	3 (20)
*ZNF442*	19p13.2	4 (26)	6 (40)
Grand Total		300	176

The most frequently deleted genes in our samples were *EPHB6, EXOC4 (SEC8L1), GNAS, MLL3 and TBX22*. The most frequently amplified genes were *HAPLN1, ADAM29, SMAD2 and SMAD4*. Gene names are according to the official HUGO Gene Nomenclature Committee and the old names in parentheses are from Sjöblom et al.

### Comparative Analysis of aCGH Data between AAs and Caucasians

We compared our aCGH data of AA patients with the list of most deleted or amplified genes obtained with Caucasian tumor tissues by Lassmann et al. [22]. The colorectal cancers in our study were more than 90% moderately differentiated which is similar to the Lassmann et al study. However, the proportions of stage II and III tumors were 68% and 32% in Lassmann et al and 33%, 53% in this study. Our comparison revealed that 29 genes have a similar pattern of alterations in both populations while 13 genes displayed different profiles (Table 4). Of these 13 genes, the *ATM* gene was mainly amplified in Caucasians. The *DCC* gene was mainly amplified in Caucasians but deleted in AAs (p<0.05). *EGR2, FLII, LLGL1, MAP2K5, PCNT, RAF1, SP6, THRB*, and *TOP3A* genes were deleted in Caucasians but unaltered in AA patients. Six genes were deleted in AAs but not in Caucasians with statistically significant differences namely; *ATM, INS, KAL1, LRRC32, TOP3A* and *XIST* (Table 4). The *STS* gene was deleted in Caucasians (p<0.05) and amplified in AAs while the *PRPF6* was amplified in AAs and unaltered in Caucasians (Table 4). The method of comparison precluded identifying genes that are frequently altered in AAs, but not included in the Lassmann et al study.

**Table 4:** Distribution of TSG and oncogenes aberrations in African Americans and Caucasians reported by Lassmann et al (22).

		Lassmann et al.		African Americans	
Gene	Chromosome band	Amplified (%)	Deleted (%)	Amplified (%)	Deleted (%)
*THRB*	3p24.3		32	0	0
*RAF1*	3p25		14	0	0
*RFC2*	7q11.2	36		33	0
*CYLN2*	7q11.23	36		33	0
*MET*	7q31	23		33	0
*LPL*	8p22		23	0	33
*E2F5*	8q22-q21.3	36		28	0
*LPL*	8p22		23	0	33
*EXT1*	8q24.11-q24.13	32		28	0
*MYC*	8q24.12-q24.13	36		28	0
*EGR2*	10q21.3		23	0	6
*DMBT1*	10q25.3-q26.1		23	0	11
*LRRC32*	11q13.5	32		6	17
*ATM*	11q22.3	27		6	17
*INS*	11p tel	32		6	17
*BRCA2*	13q12-q13	36		22	0
*RB1*	13q14	41		22	0
*MAP2K5*	15q23		32	0	11
*SP6*	17ptel		23	6	6
*TOP3A*	17p11.2			0	33
*LLGL1*	17p12-17p11.2		36	0	33
*FLII*	17p12-17p11.2		23	0	33
*HIC1*	17p13.3		32	0	28
*CTDP1*	18q tel		45	0	22
*LAMA3*	18q11.2		14	0	17
*BCL2*	18q21.3		23	0	22
*DCC*	18q21.3	32	18	0	28
*TPD52L2*	20qtel	27		33	0
*TOP1*	20q12-q13.1	32		33	6
*TNFRSF6B*	20q13	32		33	0
*NCOA3*	20q13	32		33	6
*AURKA*	20q13	36		33	6
*CSE1L*	20q13	27		33	6
*MYBL2*	20q13.1	32		33	6
*PTPN1*	20q13.1-q13.2	23		33	6
*CYP24A1*	20q13.2	36		33	6
*ZNF217*	20q13.2	32		33	6
*PRPF6*	20q13.3	27		33	0
*PCNT*	21qtel		18	0	22
*XIST*	Xq13.2	36		22	17
*STS*	Xp22.3		23	17	17
*KAL1*	Xp22.3	36		17	17

The genes listed are the forty-two genes in the Lassmann et al. report, but in some cases the name has been changed to conform with the HUGO Gene Nomenclature Committee recommendations. Markers for microsatellites in Lassmann et al. list were dropped in this comparison.

## Discussion

To decipher the possible genetic reasons underlying the high incidence of colon cancer in AAs, we earlier conducted studies on the MSI, methylation of CAN genes and mutations of known genes such as *BRAF* and *KRAS* [24], [25], [26]. While these studies revealed some of the genetic and epigenetic specifics in this population, none have shown any striking differences between AAs and the general population. We here conducted the first analysis of the whole genome of colon tumors from AA patients with the goal of having a more comprehensive view of the genomic regions involved in colon carcinogenesis. We compared our findings with those published on Caucasian samples [22] and with a widely-publicized list of colon cancer genes [23].

An average of 20 aberrations were found in the analyzed tumors strengthening the role of chromosomal instability in colon carcinogenesis in this population with more amplification (12.13 per case) than deletions (6.73 per case) pointing towards a higher role for oncogenes activation than tumor suppressor genes deactivation in this process. The CRC alterations targeted certain chromosomes more than others. Chromosomes 7, 8, 13, 20 and X were amplified in more than 50% of the cases in this study. It is established that chromosomes 7, 8, 13 and 20 are involved in CRC through amplification [22]. Our findings suggest the additional importance of chromosome X, which was amplified in 60% of cases. Many publications refer to chromosome X containing tumor suppressor genes detected after deletions in tumors [27]. Amplification of chromosome X p and q arms occurred more frequently in the male patients (5 out of 7 (71.4%)) than the female ones (2 out of 8 (25%)). A study of Japanese CRC patients found similarly that gains on chromosome X are more prominent in male than female patients [28].

In our subgroup of 7 male patients, 3 (42.8%) displayed a deletion of a common region spanning from Yq11.223 through the centromere to the Yp11.31 band. There is debate over the possible role of the Y chromosomal losses in diseases such as acute myelogenous leukemia or whether such a process is just age related [29]. Patients 4, 7 and 10 with Y chromosomal deletions are 71, 57 and 64 years old respectively. A larger male CRC population is needed to sort out the role this chromosome might play in colon cancer.

Chromosomes on which deletions are known to be frequent in CRC are 8, 15, 17 and 18 [22]. In our group, chromosomes 4, 8 and 18 displayed deletions in more than 50% of cases while chromosomes 15 and 17 were deleted at frequencies of 33.3% and 40% respectively. Thus, by the measure of chromosomes with frequent deletions, CRC in AAs appears to be similar to CRC in Caucasians.

We checked the list of 68 genes from Sjöblom et al [23] that are potentially involved in colon cancer to see the status of those genes in our group of patients. All of these genes show some level of alteration (amplification or deletion) in our patients. These results strengthen their CAN gene status (Table 3). One of the most deleted genes was *EPHB6* that is known to slow breast cancer cell lines invasiveness [30]. Another gene *EXOC4 (SEC8L1),* which contains a polymorphism associated with type 2 diabetes [31] was also frequently deleted in our samples, and is known to play a role in synaptogenesis and brain development [32]. The protein EXOC4 is part of the exocyst complex that has been implicated in breast cancer invasiveness [33]. The *MLL3* gene is not altered in Korean CRC patients [34] and rarely altered in another study [35], but it is one of the most frequent targets of deletion in our group of patients. The *GNAS* gene whose expression increases Galphas expression is a proapoptotic gene involved in many solid organ cancers [36]. Its function is consistent with our finding that it is also highly deleted in AA CRC patients. Mutations in the frequently deleted gene *TBX22* are linked to non-syndromic cleft palate [37], but *TBX2* has no known role in tumorigenesis.

Among the CAN genes from Sjöblom et al. list [23], the following are among the most frequently amplified in AA patients: *SMAD2, SMAD4, ADAM29*, and *HAPLN1*. Proteins of the ADAM family are a group of metalloproteinase of which ADAM17 is the most studied. ADAM17 is required for the generation of the active forms of Epidermal Growth Factor Receptor (EGFR) ligands, and its function is essential for the development of epithelial tissues [38]. Should ADAM29 also function to activate growth receptors, then its amplification in tumors would make sense. The *HAPLN1* (*CRTL1*) gene encodes an extracellular matrix protein, that plays an important role in heart development [39] **.** The expression of *HAPLN1* may be altered during colorectal carcinogenesis [40]. Both *SMAD2* and *SMAD4* are known to be involved in cell proliferation, apoptosis and differentiation through the TGF pathway [41]. As such, their amplification might be instrumental in growth promotion and carcinogenesis along with other genes.

We checked 42 CRC genes suggested by Lassmann et al. to be frequent targets of CNVs [22]. We comment on a few of the genes for which the Caucasian and AA samples showed different patterns of aberrations. The *ATM* gene, whose encoded protein is essential for DNA damage response and contributes to cellular homeostasis [42], was frequently amplified in Caucasian patients but not in the AA group. On the other hand, *PRPF6* is only amplified in AAs (Table 4). Mutations in genes from the same family (*PRPF3*, *8*, and *31*) have been implicated in retinitis pigmentosa [43]. However, no role in cancer of *PRPF6* or other related genes is known. Two genes showed opposite alterations in the two patient groups; *DCC* was primarily amplified in Caucasians but deleted in AAs, while the *STS* gene was deleted in Caucasians and amplified in AAs. Indeed, *DCC* is generally down-regulated or deleted in colon cancer patients owing to its TSG properties which is more consistent with its deletion status using aCGH in our patients [44]. The constitutive expression of *STS* gene (steroid sulfatase gene) promotes the growth of human breast cancer cells [45]. While the differences between Lassmann et al, Sjöblom et al [22], [23] and our study might be in part due to the level of chromosomal aberration, the data within our study showed that neither the tumor stage nor the differentiation status had an effect on chromosomal instability. Future studies are needed where stage and differentiation matched tumors from different populations are evaluated.

In conclusion, our aCGH analysis of 15 AA colorectal carcinomas shows that all tumors contain some level of chromosomal instability. Georgiades et al. have identified a group of carcinomas with no CIN [4]. Such is not the case for our AA patients. A larger number of patients is needed to investigate whether such CRC tumors exist within AAs or all AA CRCs have some levels of CIN. A more comprehensive analysis involving MSI and methylation profiles of the analyzed tumors, in addition to CIN analysis might also shed more light on the intricacies and specificities of these different processes in tumorigenesis. The role of Chromosome X amplification in colon carcinogenesis in AA patients, particularly males, merits further investigation.
